# *Mycobacterium indicus pranii* Induced Memory T-Cells in Lung Airways Are Sentinels for Improved Protection Against M.tb Infection

**DOI:** 10.3389/fimmu.2019.02359

**Published:** 2019-10-18

**Authors:** Ananya Gupta, Mohd Saqib, Bindu Singh, Lalit Pal, Akoijam Nishikanta, Sangeeta Bhaskar

**Affiliations:** ^1^National Institute of Immunology, Product Development Cell-I, New Delhi, India; ^2^Department of Immunology and Microbial Disease, Albany Medical College, Albany, NY, United States

**Keywords:** airway lumen, chemokine receptor, memory T-cell, *Mycobacterium indicus pranii* (*Mw/*MIP), route of vaccination

## Abstract

The lungs are the most vulnerable site for air-borne infections. Immunologic compartmentalization of the lungs into airway lumen and interstitium has paved the way to determine the immune status of the site of pathogen entry, which is crucial for the outcome of any air-borne infections. Vaccination via the nasal route with *Mycobacterium indicus pranii* (MIP), a prospective candidate vaccine against tuberculosis (TB), has been reported to confer superior protection as compared to the subcutaneous (s.c.) route in small-animal models of TB. However, the immune mechanism remains only partly understood. Here, we showed that intranasal (i.n.) immunization of mice with MIP resulted in a significant recruitment of CD4^+^ and CD8^+^ T-cells expressing activation markers in the lung airway lumen. A strong memory T-cell response was observed in the lung airway lumen after i.n. MIP vaccination, compared with s.c. vaccination. The recruitment of these T-cells was regulated primarily by CXCR3–CXCL11 axis in “MIP i.n.” group. MIP-primed T-cells in the lung airway lumen effectively transferred protective immunity into naïve mice against *Mycobacterium tuberculosis* (M.tb) infection and helped reducing the pulmonary bacterial burden. These signatures of protective immune response were virtually absent or very low in unimmunized and subcutaneously immunized mice, respectively, before and after M.tb challenge. Our study provides mechanistic insights for MIP-elicited protective response against M.tb infection.

## Introduction

Tuberculosis continues to be a major threat to human health worldwide. The etiological agent, M.tb, has evolved in a manner to modulate the host immune response in its favor. Its complex cell-wall and secretome interferes with innate immune signaling pathways of the host, which culminates in a delayed onset of adaptive response at the primary site of infection. This delayed immune activation favors an exponential growth of M.tb and the successful establishment of infections (1).

Vaccine approach and the strategy of immunization play a crucial role in prevention against infectious diseases like TB. The whole bacterial vaccine approach has the advantage of multiple antigens and is built in adjuvanticity. In addition to a better vaccine approach, the vaccine administration route is important to obtain site specific long lasting immunity. In case of infectious diseases like pulmonary TB, a strong local lung immunity seems extremely logical for an overall systemic immunity. The i.n. route of vaccination directly targets the most vulnerable compartment of lungs i.e., the airway lumen. This could result in early adaptive immune cell activation and its recruitment to the desired location (airway lumen); which is believed to be critical for protection against air-borne pulmonary infections such as TB (2).

Since its development as an anti-leprosy vaccine during 1990s, *Mycobacterium indicus pranii* (MIP) has been evaluated successfully as prophylactic as well as therapeutic vaccine against TB in animal models and in clinical set-ups. Beside the presence of its own unique immunogens, MIP also shares a huge repertoire of highly antigenic PE/PPE proteins of M.tb that renders it as a promising vaccine candidate (3, 4). Preclinical studies using M.tb-challenge models have compared the protective efficacy of nasal and subcutaneous route of MIP vaccination. Although, MIP given by subcutaneous route reduces M.tb burden in the lungs, but nasal delivery of MIP further lowers the bacterial burden and results in improved pulmonary pathology (5–7).

The objective of this study was to assess the lung immune response in the two different compartments when MIP was given via i.n. route in comparison to parenteral (s.c.) route. We hypothesized that i.n. MIP mediated accumulation of mycobacterium-specific lung resident T cells that leads to improved protection against incoming M.tb infection. Indeed, we found that i.n. vaccination with MIP elicited strong CD4^+^ and CD8^+^ T-cell responses as well as robust T-helper 1 (Th1) recall response in lung airway lumen. These phenomena correlated with significantly greater protection observed in previous studies as compared to s.c. immunization. Importantly, the memory response thus elicited in the airway lumen could adoptively transfer protection to naïve mice challenged with M.tb. Because of their strategic location and rapid recall response, alveolar memory T-cells represent preferred cellular targets for an efficacious vaccination. Thus, the route of MIP vaccination matching the route of pathogen entry proffers an immunologically advantageous position over the conventional route.

## Materials and Methods

### Ethical Approval of the Study Protocol

The study protocol was approved by the Ethics Committee of the National Institute of Immunology (New Delhi, India). Experimental procedures were in accordance with the guidelines of Animal Ethics and Bio-safety Committee of the National Institute of Immunology.

### Animals and Bacteria

Inbred female C57Bl/6 mice (6–8 weeks) from the National Institute of Immunology, were maintained in pathogen free conditions. *Mycobacterium tuberculosis* (H37Rv strain) and *Mycobacterium indicus pranii* (MIP) were grown in 7H9 media supplemented with 10% Albumin Dextrose Catalase (ADC), 0.2% glycerol and 0.05%/0.1% tween-80 for MIP/M.tb-H37Rv, respectively. Culture was harvested at mid-log phase.

### Immunization and Infection in Mice

Mice were divided into three groups: Control, MIP i.n., MIP s.c. The MIP groups were immunized with live MIP via the i.n. and s.c. routes, respectively, twice at an interval of 3 weeks. The control group received saline via the intranasal route. For i.n. immunization, anesthetized mice were inoculated with 1 × 10^6^ CFU in ~50 μl PBS into the nostril using 24 G tubing which resulted in ~1,000 CFU in the lungs as determined by counting of CFUs on 7H11 culture plates. For s.c. immunization, 5 × 10^6^ CFU of MIP in 100 μl PBS was injected just beneath the skin, at right flank near lower limb.

To determine protective efficacy, mice were challenged with 200 CFU of aerosolized M.tb-H37Rv 1 day post-adoptive transplantation of T-cells by using a Madison inhalation exposure system (Madison industries, USA).

### Immunohistochemistry of Lungs

Non-perfused lungs from unvaccinated and MIP-vaccinated mice were fixed in 4% (wt/vol) paraformaldehyde for 24 h and then dehydrated and embedded in paraffin for analysis. Sections (4 μm) were taken on glass slides, deparaffinized, and subjected to immunofluorescence. Tissue sections were stained with antibodies against T-cells (anti-mouse CD3; Abcam, Cambridge, UK) and B-cells (biotin-conjugated B220; BioLegend, San Diego, CA, USA). The secondary antibodies used were anti-rabbit Alexa Fluor™ 488 and streptavidin-conjugated Alexa Fluor 594 (Life Technologies, Carlsbad, CA, USA). Slides mere stained with DAPI (Sigma–Aldrich, Saint Louis, USA) and mounted using ProLong® Gold mounting reagent (Invitrogen, Carlsbad, CA, USA).

### Isolation of Cells

Mice were euthanized by an overdose of anesthesia (Ketamine-Xylazine cocktail). To prepare airway luminal cells (also referred to as BAL cells) bronchial lavage was undertaken. Briefly, lungs and trachea of mice were exposed by making incisions. Lungs were flushed 4-5 times with chilled PBS-EDTA (5 mM- EDTA in 1x PBS) using a 24 G catheter inserted into the trachea of mice. For preparation of lung interstitium cells, lungs devoid of BAL cells were collected and minced into small pieces (1 mm^2^). Tissue was digested by incubating tissue in an enzyme cocktail- collagenase A (0.7 mg/ml), DNase (30 μg/ml), and hyaluronidase (0.2 mg/ml) at 37°C for 45 min with continuous shaking. Digested lung tissue was then forced through cell-strainer (pore size 40 μm); extracellular matrix was excluded and single cells of the interstitium were obtained in suspension. Spleens collected from euthanized mice were crushed using frosted-end slides to prepare single cell suspension. RBCs were lysed using freshly prepared Gey's buffer solution.

### Estimation of Secretory Cytokines and Lymphocyte Proliferation Assay (LPA)

1 × 10^6^ cells of each compartment (lung airway lumen and lung interstitium) were plated in RPMI-10 (RPMI + 10% FBS + 1% antibiotic cocktail) in 24-well tissue culture plate and stimulated with M.tb whole cell lysate. Culture supernatants were collected after 24 and 48 h of incubation at 37°C. Cytokine levels were measured by ELISA.

For the LPA, 0.2 × 10^6^ cells from the above compartments were plated in 96-well-round bottom plates and stimulated with M.tb whole cell lysate. Following 72 h of incubation, cultures were pulsed with 1 μCi of [^3^H]-thymidine and incubated for an additional 18 h. Thymidine uptake by proliferating cells was measured by a scintillation counter.

### Intracellular Cytokine Staining and Flow Cytometry

For intracellular cytokine staining, cells of lung airway lumen and interstitium were stimulated *ex-vivo* with 20 μg/ml of M.tb whole cell lysate in complete RPMI medium for 18 h at 37°C. Cells were cultured for an additional 6 h with GolgiStop™ (BD Pharmingen, Franklin Lakes, NJ, USA). Following stimulation, cells were harvested and washed with FACS buffer (PBS + 1% Bovine Serum Albumin) and incubated with Fc receptor blocking antibody (CD16/CD32 mAb). Then, cells were stained for 30 min at 4°C for surface markers- CD4 (FITC)/CD8 (eFluor 450), washed in FACS buffer, permeabilized using Cytofix/Cytoperm™ kit (BD Pharmingen) according to manufacturer's instructions and stained intracellularly for 30 min at 4°C for cytokines—IFN-γ (PE), TNF-α (PE-Cy7), and IL-2 (APC).

For surface staining, 0.5 x 10^6^ cells of each compartment i.e., lung airway lumen, lung interstitium, and spleen were taken. The cells were washed twice with FACS buffer (PBS + 1% BSA), were incubated for 30 min at 4°C with fluorescent labeled antibodies against surface markers—CD4 (FITC)/CD8 (eFluor 450)/CD14 (PerCP5.5)/CD11c (PE-Cy7) and Nk1.1 (APC). Following staining, cells were subsequently washed twice, re-suspended in FACS buffer and analyzed in FACSVerse™ (BD Biosciences, San Jose, CA, USA).

### Gene Expression Analysis

Expression of chemokine markers in lungs of respective groups of mice were determined by measuring mRNA copy number using qRT-PCR. Whole lung RNA was prepared using One Step RNA Reagent (Bio Basic, Markham, ON, Canada) as per manufacturer's instruction. mRNA was quantified by spectrophotometer and the quality was examined by agarose gel electrophoresis. cDNA was prepared from 1 μg RNA using Verso™ cDNA kit (Thermo Scientific, Waltham, MA, USA) according to manufacturer's recommendations. mRNA levels of different cytokine genes were measured using a RT-PCR mix (HOT FIREpol®) and the samples were run in Master Cycler™ Real Plex (Eppendorf, Hamburg, Germany). Relative quantification of genes was done by 2^−CtΔΔ^ method.

### Adoptive T-Cell Transfer

Airway luminal cells were collected from mice immunized with MIP via i.n. route. Cells were sorted using negative selection CD4^+^ and CD8^+^T lymphocyte-enrichment kit (BD IMag™) as per manufacturer's protocol. Briefly, BAL cells were incubated with a biotin-labeled antibody cocktail followed by streptavidin-labeled magnetic beads at 4°C. The cocktail was subjected to magnetic field and the suspension was collected separately. This step was repeated twice to ensure maximum enrichment. Enriched cells were analyzed for their respective purity by FACS.

Mice were anesthetized (using 80 mg Ketamine and 8 mg Xylazine cocktail per kg body weight of mice) to an unconscious state and fixed on vertical platform gently. 5 × 10^4^ sorted airway luminal CD4^+^ and CD8^+^ T-cells suspension in PBS were transplanted into mice trachea using intubation tube of 1 mm diameter and the procedure was performed inside laminar hood (Figure 10A).

### CFU Enumeration

At designated time points after infection in mice, whole lung was homogenized in 1 ml of PBS by using homogenizer (Polytron® PT 1600; Kinematica, Lucerne, Switzerland). One hundred microliter of 10-fold serial dilutions of lung homogenates were plated on 7H11 agar plates in triplicates. The plates were incubated at 37°C for 3–4 weeks, and colonies were counted manually.

### Statistical Analysis

Statistical analyses were undertaken using Prism7 (GraphPad, San Diego, CA, USA). Data from independent experiments were pooled and plotted as standard error of the mean (SEM). *p* ≤ 0.05 was considered significant.

## Results

Success of a vaccine lies in its ability to induce tissue resident and systemic antigen-specific memory response. In this light, we evaluated differential ability of i.n. and s.c. routes of vaccination in eliciting protective immune cells in different lung compartments and spleen of mice.

### Intranasal MIP Vaccination Induced Pulmonary Localization of Immune Cells

**Figure 1:**
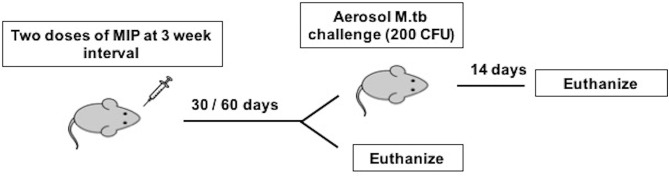
Immunization and infection scheme of C57Bl/6 mice. Animals were given two doses of MIP and sacrificed at 4 and 8 weeks after MIP booster for before infection studies. The animals challenged with M.tb following 4 (Group 1) and 8 (Group 2) weeks of vaccination were euthanized 14 days post-infection.

To ascertain the effect of intranasal MIP immunization on pulmonary immune cell profile, airway luminal cells were collected from immunized (i.n./s.c.) and an unimmunized control groups and analyzed (Figure 1). Although the total percentage of leukocytes remained comparable, increase and decrease in frequency of different immune cell populations were observed in the airway lumen of all the three groups. Total leukocytes in lung interstitium (which is devoid of airway luminal cells) and spleen, were also similar before and after immunization (Figure S1). This suggests that MIP immunization does not elicit an untoward inflammatory response in any of the body compartments.

### Intranasal MIP Immunization Leads to Differential Influx of CD4^+^ and CD8^+^T-Cells Into the Airway Lumen of Lungs

**Figure 2:**
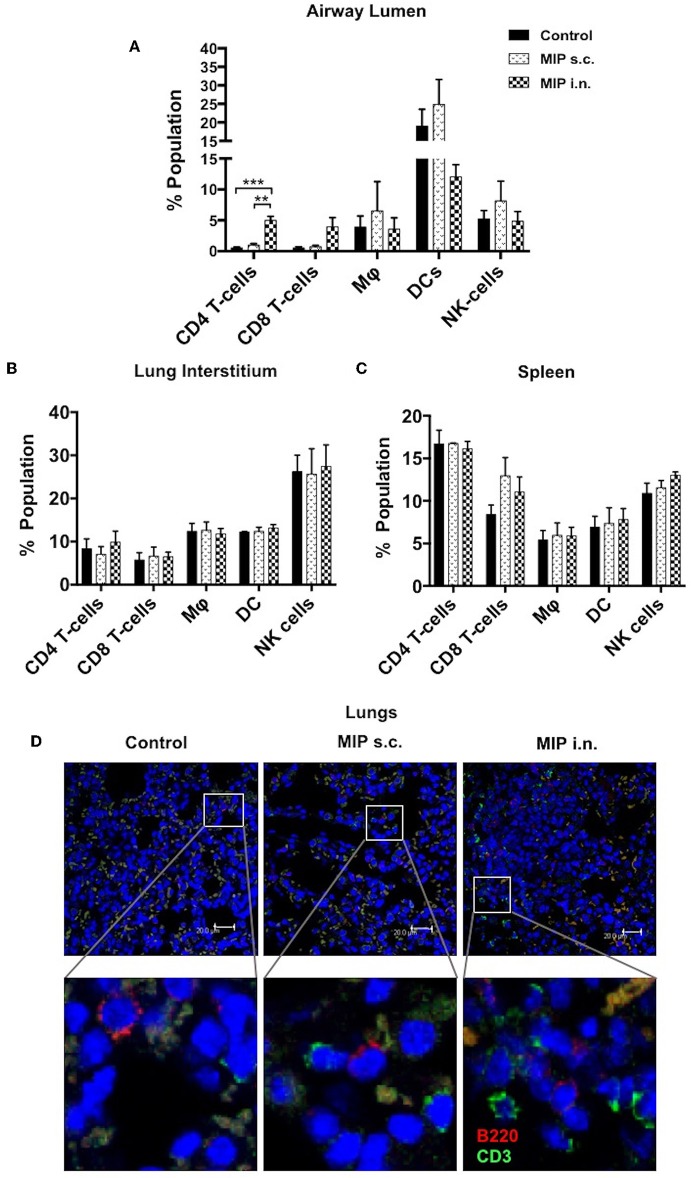
Immune cell composition 4 weeks post-immunization. **(A)** Bronchalveolar lavage fluid (BALF) cells isolated from airway lumen were analyzed by FACS using surface markers for T-cells, macrophages, DCs and NK-cells. **(B,C)** Immune cell composition 4 weeks post-immunization in lung interstitium (lacking BAL cells) and in spleen. Data are mean ± SEM; analysis was done using two-way ANOVA with Tukey's correction for multiple comparisons. **p ≤ 0.002 and ***p ≤ 0.0008 (*N* = 3, 8–10 mice/group/experiment). *N*, number of experiments. **(D)** Representative picture of immunohistochemistry (non-quantitative) of lung section of MIP immunized vs. unimmunized mice showing T-cells (CD3+ -Green) and B-cells (B220+ -Red) infiltration.

To determine the status of various immune cells in lung airways, interstitium and spleen before and after MIP immunization, cells of these lung compartments were analyzed for frequency of T-cells, macrophages, dendritic cells, and natural killer cells. Following MIP i.n. vaccination, the immune equilibrium of airway lumen was found to be shifted toward T-cells. There was ~5-fold increase in the percentage of CD4^+^ (*p* < 0.002) and CD8^+^ T cells in the airway lumen 4 weeks after “MIP i.n.” vaccination as compared to “MIP s.c.” vaccination (Figure 2A). A similar increase in the proportion of T-cells between the two groups was also observed at 8 weeks (data not shown). Similar to findings of flow-cytometric analysis, a non-quantitative immunohistochemistry analysis also showed higher number of CD3^+^ cells which in the lung section of “MIP i.n.” group as compared to “MIP s.c.” group; these cells were uniformly distributed throughout the lung sections. Frequency of B-cells in lung section of all the three groups was found to be similar (Figure 2D). A simultaneous reduction in the percentage of innate immune cells was observed (specifically DCs) in the airway lumen compartment (Figure 2A). But interestingly, no significant difference in the percentage of these T-cell subsets could be observed between the two groups, in the lung interstitium (Figure 2B). Importantly, both routes of MIP delivery induced an increase in CD8^+^ T-cells in spleen as compared to control; this corresponds with our previous studies where a good CD8^+^ T-cell response was always observed with MIP vaccination (Figure 2C). These results suggest that intranasal MIP vaccination drives localization of T-cells at the primary site of M.tb infection as well as in the systemic compartment.

### T_EM_ and T_CM_ Cells Generated in Lung Airway Lumen After MIP Vaccination

**Figure 3:**
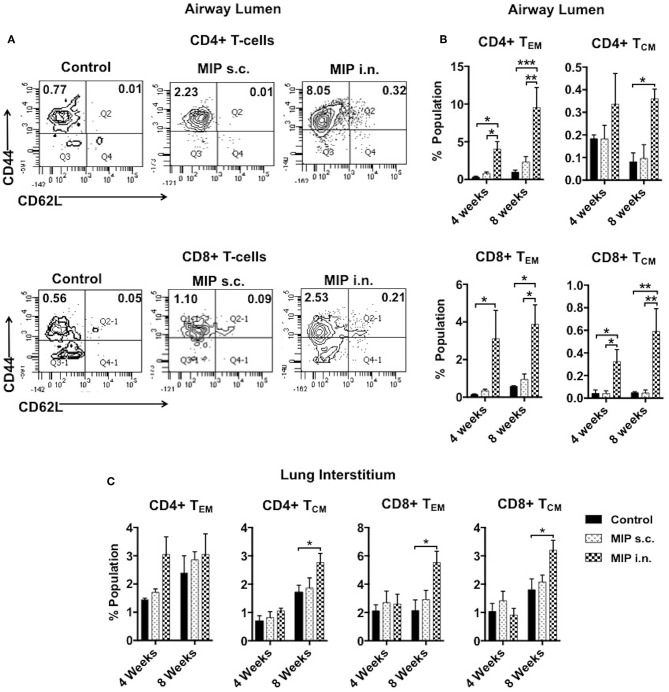
Memory T-cell subpopulations in lung. **(A)** Representative FACS profile for effector (CD44+ CD62L–) and central (CD44+ CD62L+) memory T-cells 8 weeks post-i.n. immunization in lung airway lumen. **(B)** Mean of three sets showing effector and central memory T-cells (both CD4 and CD8) at 4 and 8 weeks after vaccination in airway lumen and **(C)** lung interstitium. Data are mean ± SEM; analysis was done using two-way ANOVA with Tukey's correction for multiple comparisons. *p ≤ 0.05, **p ≤ 0.005, and ***p ≤ 0.0009 (*N* = 3, 8–10 mice/group/experiment). *N*, number of experiments.

The significant increase in T-cell numbers in airway lumen following MIP i.n. vaccination encouraged us to further investigate various memory T-cell subpopulations. Cells isolated from lung airway lumen after 4- and 8-weeks of immunization were analyzed for the proportion of T_EM_ (CD44^+^ CD62L^−^) and T_CM_ (CD44^+^ CD62L^+^) cells in both CD4^+^ and CD8^+^ T-cell subsets. The proportion of CD4^+^ and CD8^+^ T_EM_ cells in the airway lumen of “MIP i.n.” group as compared to “MIP s.c.” group were ~5 and ~9-folds higher, respectively. Although the overall proportion of T_CM_ in this peripheral compartment was ~10-fold less than T_EM_ cells; the percentage of CD8^+^ T_CM_ was significantly higher (*p* < 0.04; 4 weeks, *p* < 0.005; 8 weeks) in “MIP i.n.” group as compared to “MIP s.c.” group. A similar trend was also observed in CD4^+^ T_CM_ cells in this lung compartment (Figure 3B). The differential response observed between these two groups in the airway lumen compartment at 4 weeks after immunization, was present even after 8 weeks. These results demonstrated the limited ability of s.c. vaccination in mounting a local lung memory response even at later time points. Lung interstitium and spleen were also analyzed. There was no significant difference in the percentage of memory T-cells at 4 weeks; whereas at 8 weeks the frequency of T_CM_ (both CD4 and CD8 subsets) was observed to be significantly higher in the lung interstitium of “MIP i.n.” group as compared to “Control” group (*p* < 0.02) (Figure 3C). Also, q-PCR analysis of the whole lung (with intact airway luminal cell population) isolated 4 weeks after vaccination revealed a significantly higher expression of Ki67 in the “MIP i.n.” group as compared to “MIP s.c.” group (*p* < 0.04) (Figure S2), suggestive of a higher proportion of actively dividing cells making their way into airway luminal compartment post-MIP i.n. vaccination.

In spleen, MIP immunization by both routes induced similar level of central memory CD8^+^ T-cells which was higher than “Control” group (Figure S3), highlighting the advantage of i.n. route over parenteral route in inducing robust systemic response besides inducing potent local lung response.

### MIP Induces Th1 Type of Cells in Lungs

**Figure 4:**
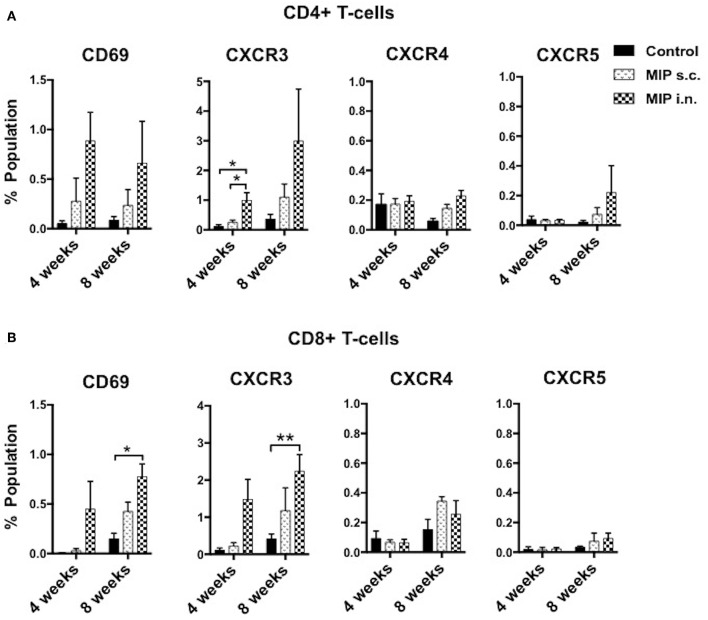
Phenotype of T-cells in the airway lumen following immunization. **(A,B)** CD4^+^ and CD8^+^ T-cells of airway lumen were stained for markers CXCR3, CXCR4, and CXCR5 and activation marker CD69 at 4 and 8 weeks post-immunization. Data is mean ± SEM of three independent set of experiments; analysis was done using two-way ANOVA with Tukey's correction for multiple comparisons. *p ≤ 0.02 and **p ≤ 0.006 (*N* = 3, 8–10 mice/group/experiment). *N*, number of experiments.

**Figure 5:**
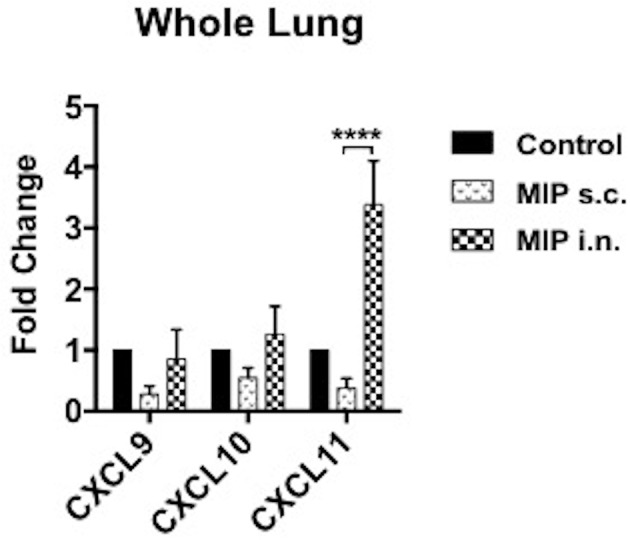
Expression of chemokines in lungs. mRNA expression of ligands for CXCR3 receptor were evaluated in lungs of mice 4–5 weeks after MIP immunization. RT-PCR for chemokines CXCL9, CXCL10, and CXCL11 were performed. Data are mean ± SEM of 8 mice in each group from two independent set of experiments. Analysis was done using two-way ANOVA with Tukey's correction for multiple comparisons where ****p ≤ 0.0001.

Antigen stimulated T-cells, beside their memory phenotype, are characterized for expression of the migration markers that also define their functional activity. CXCR3 is a chemokine receptor which helps migration of Th1 cells into the lungs (8, 9). In lung airway lumen, ~3-folds more number of CD4^+^ CXCR3^+^ T-cells and ~6-folds more CD8^+^ CXCR3^+^ T-cells were observed in the “MIP i.n.” group as compared to the “MIP s.c.” group, suggesting effective migration of these cells in the airway lumen of the i.n. immunized group (Figures 4A,B). To assess the involvement of cognate ligand(s) for CXCR3 receptor (which facilitates its migration to lung airways following MIP vaccination) mRNA expression of CXCL9, CXCL10, and CXCL11 were measured in whole lungs of mice 4–5 weeks after MIP immunization. High induction of the chemokine ligand CXCL11 was observed in the lungs in response to MIP i.n. immunization, which was not found in “MIP s.c.” group. The group also displayed a moderate increase in CXCL10 expression (Figure 5). Proportions of CXCR4 and CXCR5 expressing Th2 type cells, which help in the development and recruitment of B-cells, were found to be low and comparable after MIP administration by both routes (Figures 4A,B). These observations provide evidence that the majority of T-cells in the lungs were of Th1 type following immunization with MIP.

CD69 is expressed on T-cells following activation by antigen-MHC complex. The effect of MIP immunization on recruitment of CD69^+^ T-cells in lung compartments was analyzed. At 4 weeks after immunization, ~3-folds higher frequency of CD4^+^ CD69^+^ and ~15-folds higher frequency of CD8^+^ CD69^+^ T-cells were observed in the airway luminal compartment of “MIP i.n.” group as compared to “MIP s.c.” group. The difference also persisted at later time points i.e., 8 weeks post-vaccination (Figures 4A,B and Figures S6A,B).

### M.tb Antigen Specific Memory Recall Response

**Figure 6:**
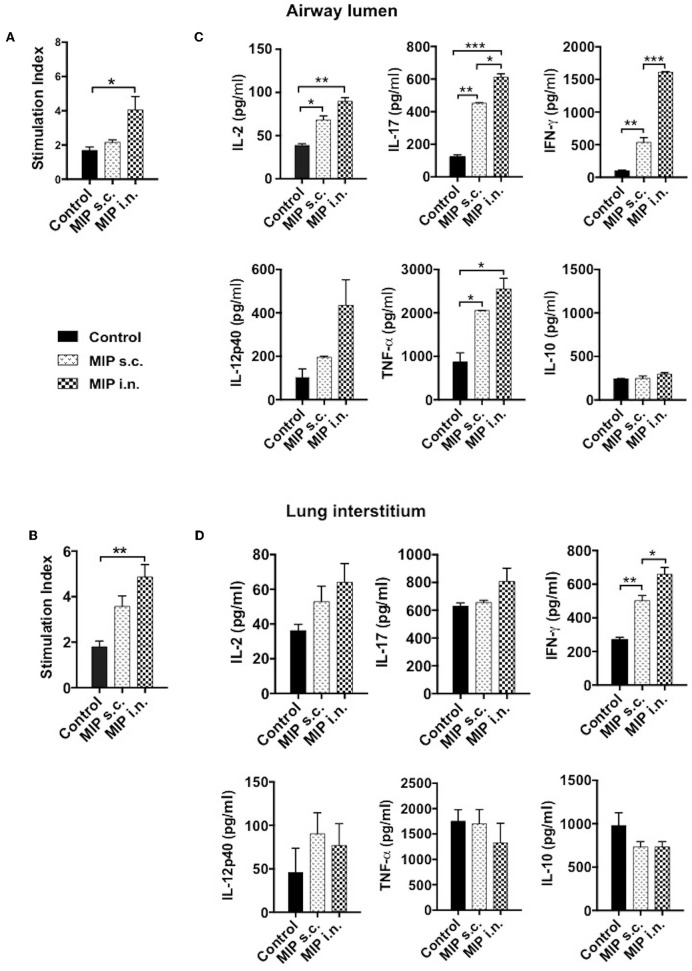
Memory recall response. Proliferation potential of T-cells were analyzed, cells of **(A)** lung airway lumen and **(B)** interstitium isolated from 8 to 10 mice were pooled separately and re-stimulated with M.tb whole cell lysate for 72 h. Cells were then pulsed with thymidine [^3^H] for additional 18 h to analyse its proliferation potential. **(C,D)** Cells from lung airway lumen and lung interstitium were incubated with M.tb whole cell lysate and supernatants were collected 24 and 48 h after treatment. Proinflammatory cytokines-IL-2, IL-17, IFN-γ, IL-12p40, TNF-α, and anti-inflammatory cytokine IL-10 were analyzed by ELISA. Data are mean ± SEM and analysis was done using one-way ANOVA with Tukey's correction for multiple comparisons. *p ≤ 0.03, **p ≤ 0.007, ***p ≤ 0.0005 (*N* = 2, 8–10 mice/group/experiment). *N*, number of experiments.

We intended to assess the memory recall response of MIP primed T-cells in lung compartments *ex-vivo*. This was measured by their ability to proliferate in response to M.tb whole cell lysate, 4–5 weeks post-MIP immunization. Approximately 2-fold higher proliferation was observed in the airway luminal cells isolated from “MIP i.n” group as compared to “MIP s.c.” group (Figure 6A). Lung interstitium cells from “MIP i.n.” group also displayed higher proliferative capacity toward M.tb antigen stimulation as compared to s.c. immunized group (Figure 6B). The result showed that following s.c. vaccination, only limited number of MIP-primed cells could migrate to the lung via circulation due to absence of specific homing markers and only a tiny percentage could egress out in the airway lumen.

### MIP Primed Immune Cells Produce Th1 and Th17 Type Cytokines

Phenotypic analysis clearly demonstrated the significant presence of helper T-cells in the airway lumen of immunized mice in comparison with unimmunized mice. The key effector populations orchestrating the protective and pathological response in TB are Th1, Th17, and Th2 cells (10, 11). These populations have their own distinct cytokine profile. Total cells from lung airways, interstitium and spleen were isolated 4 weeks post-vaccination; analyzed for pro- and anti-inflammatory cytokines secretion, *ex-situ*, in response to M.tb antigen, by ELISA. Cells isolated from lung airways lumen produced Th1 and Th17 cytokines in response to M.tb antigens. Memory T-cells from the airway lumen of “MIP i.n.” group secreted significantly high amounts of IFN-γ (*p* < 0.0005) and IL-17 (*p* < 0.03), compared with the “MIP s.c.” group. Secretory level of the other Th1 cytokine IL-2 was relatively high (*p* = 0.2072) in “MIP i.n.” group though not significantly, while the levels of TNF-α, IL-12, and IL-10 remained similar across the groups (Figure 6C). Cells isolated from lung the interstitium of “MIP i.n.” group secreted significant amounts of IFN-γ (*p* < 0.03) whereas moderate amounts of IL-17 and IL-2 were observed in the culture supernatant (Figure 6D). Interestingly, the response of splenocytes from “MIP i.n.” group was quantitatively similar to that of s.c. immunized group. Comparable secretions of IFN-γ and TNF-α were observed in “MIP s.c.” and “MIP i.n.” groups in this compartment providing evidence of the systemic response also besides potent lung immune response induced by intranasal immunization (Figure S4). IL-2 secretion was observed from immune cells of all the compartments post-immunization which correlates with higher frequencies of central memory T-cells observed by FACS analysis after MIP immunization (Figure 3 and Figure S3).

### Comparison of M.tb-Ag Specific Polyfunctional T-Cells in Lung Airways

MIP induced memory T-cells were evaluated for their ability to produce more than one type of cytokine per cell. Reports of T cells simultaneously producing IFN-γ, TNF-α, and IL-2 or a combination of any two, underpins their crucial role in protection against TB (12–14). T-cells secreting more than one cytokine have been reported to be functionally superior and comparatively more efficient than their single cytokine producing counterparts (15, 16).

**Figure 7:**
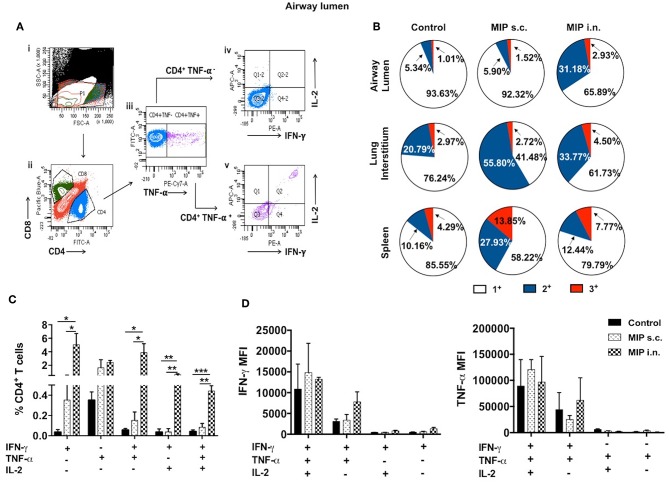
Polyfunctional CD4^+^ T-cells. **(A)** Representative plot shows the gating strategy for analysis of cytokine producing CD4^+^ T-cells. (i) Lymphocyte region (P1) was gated on SSC vs. FSC plot, (ii) P1 region was drilled down to obtain CD4^+^ and CD8^+^ populations. (iii) CD4^+^ population positive and negative for TNF-α were further gated for IFN-γ and IL-2 secretion (iv, v). **(B)** Pie-chart showing 3^+^/2^+^/1^+^ cells out of total responder CD4^+^ T-cells in pulmonary and splenic compartments. **(C)** CD4^+^ T cells from airway lumen secreting all three cytokines (3+), any two cytokines (2+) or any one cytokine (1+) were assessed by intracellular staining with antibodies specific to IFN-γ, TNF-α, and IL-2. Four weeks post-MIP immunization, cells from airway lumen stimulated *ex-vivo* with M.tb whole cell lysate for 24 h. **(D)** Mean fluorescence intensity (MFI) of IFN-γ and TNF-α in 1+, 2+, and 3+ T-cell populations in the airway lumen of different groups of mice. Data are mean ± SEM; analysis was done using two-way ANOVA with Tukey's correction for multiple comparisons. *p ≤ 0.02, **p ≤ 0.005, and ***p ≤ 0.0005 (*N* = 3, 10–12 mice/group). *N*, number of experiments.

It was observed that MIP primed T-cells, that were responsive toward M.tb whole cell lysate, secreted one or more of pro-inflammatory cytokines—IFN-γ, TNF-α, and IL-2 (Gating strategy shown in Figure 7A). Total percentage of M.tb responsive CD4^+^ T-cells were obtained by summing up the actual percentage of 3+ (all three cytokines producer), 2+ (any two of the above mentioned cytokines producer), and 1+ (either of the 3 cytokines producer) population. Pie-chart analysis revealed the percentage distribution of 3+, 2+, and 1+ populations within total M.tb-responsive CD4^+^ T-cells in the three compartments of “unimmunized control,” “MIP s.c.,” and “MIP i.n.” group. Major proportions of these cells from all the three compartments of “unimmunized control” group were found to secrete only one type of cytokine. Immunization with MIP (by both routes) potentiated the cells to produce more than one cytokines; MIP i.n. vaccination induced about 2-folds higher 3+ populations in both lung compartments as compared to MIP s.c. vaccination, but vice-versa was true for splenic compartment. It was also noted that MIP s.c. vaccination induced higher proportion of 2+ CD4^+^ T-cells in lung interstitium (but not in lung airways) and spleen as compared to nasal route of MIP vaccination (Figure 7B). These observations suggested that location of stimulus decides the quality and magnitude of immune response in the vicinity and at sites far from it.

Detailed analysis revealed that a significantly high percentage of 3+ (IFN-γ^+^ TNF-α^+^ IL-2^+^ with *p* = 0.0014) CD4^+^ T-cells were observed in the airway lumen of “MIP i.n.” group, compared with “MIP s.c.” group. Beside 3+ CD4^+^ T-cells, a significant proportion of 2+ (IFN-γ^+^ TNF-α^+^ with *p* = 0.030; IFN-γ^+^ IL-2^+^ with *p* = 0.006) CD4^+^ T-cells were present in this compartment of intranasally vaccinated group, when compared with “MIP s.c.” group. Apart from polyfunctional CD4^+^ T-cells, i.n. immunization also elicited significantly high number of IFN-γ (*p* = 0.031) producing cells, whereas, proportion of TNF-α^+^ secreting cells in the airway lumen was similar in both i.n. and s.c. groups. Though importance of polyfunctional CD4^+^ T-cells in long term protection against TB is known by now, the crucial role of monofunctional T-cells producing cytokines like IFN-γ and TNF-α cannot be denied (13). The results indicated that vaccination with MIP by nasal route correlated with higher frequency of polyfunctional as well as single cytokine secreting (IFN-γ^+^) CD4^+^ T-cells (Figure 7C). Mean fluorescence intensity of IFN-γ and TNF-α were found to be highest in 3+ cells followed by 2+ and 1+ populations (Figure 7D).

### Effector Responses in Intranasally Immunized Mice Post-M.tb Challenge

M.tb infection is known to delay the activation and recruitment of immune cells at the site of primary infection (17). This delay provides the pathogens a sufficient time window to increase their number exponentially. In this light, we studied immune response at 2 weeks post-M.tb aerosol challenge in “unimmunized control” and MIP immunized groups (challenged 4 and 8 weeks after immunization).

#### Composition of Immune Cells

**Figure 8:**
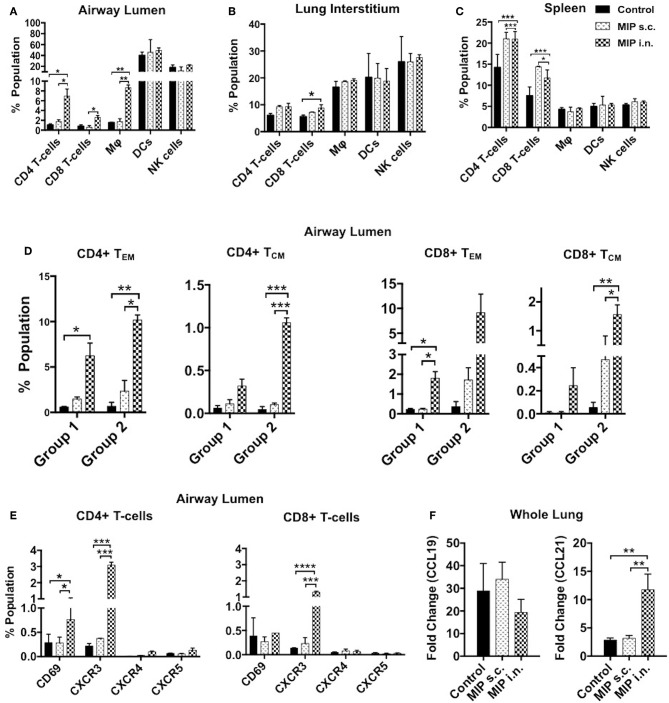
Immune response in different compartments post-M.tb challenge. T-cells, macrophages, DCs and NK-cells were analyzed with the help of their definitive surface markers 2 weeks post-M.tb infection in **(A)** lung airway lumen, **(B)** interstitium, and in **(C)** spleen of Group 1 (challenged with M.tb 4 weeks after MIP vaccination). **(D)** Proportion of effector and central memory T-cells (both CD4 and CD8) migrating into airway lumen of Group 1 and Group 2 (challenged after 8 weeks of immunization). **(E)** Proportion of airway luminal T-cells expressing chemokine receptors CXCR3, CXCR4, CXCR5, and activation marker CD69 (Group 1). Data are mean ± SEM and analysis was done using two-way ANOVA with Tukey's correction for multiple comparisons. *p ≤ 0.02, **p ≤ 0.005, ***p ≤ 0.0005, and ****p ≤ 0.0001 (*N* = 2, 10–12 mice/group). *N*, number of experiments. **(F)** Expression of chemokine ligands CCL19 and CCL21 were evaluated by qRT-PCR in lung draining lymph node after 2 weeks post-M.tb challenge. Data are mean ± SEM of 6 mice from two independent set of experiments in each group and analysis was done using one-way ANOVA with Tukey's correction for multiple comparisons. *p ≤ 0.03.

The immune cell composition of lung compartments and spleen, at 2 weeks post-M.tb infection, in immunized groups vs. only M.tb infected control group was assessed. Mice challenged with M.tb 4 weeks after MIP i.n. vaccination, showed higher percentage of CD4^+^ and CD8^+^ T-cells in the airway luminal compartment (as observed in “before infection study”). Interestingly, in contrast to “before infection study” where frequency of macrophages didn't increase following immunization, a significantly higher percentage of macrophages (*p* = 0.003) was observed 2 weeks post-infection in the airway lumen of “MIP i.n.” group as compared to s.c. group (Figure 8A). Lung interstitium had a marginal increase in the number of T-cells in MIP i.n. group, with no change in macrophages, DCs and NK-cells (Figure 8B). Significant increase in the T-cells (CD4^+^ and CD8^+^) was observed in the spleen of both the MIP immunized groups and the control group (Figure 8C). Presence of effector memory T-cells in the airway lumen as well as in the systemic compartment at 2 weeks post-M.tb infection could be responsible for a better control of the infection in the “MIP i.n.” group as compared to the “MIP s.c.” group, as observed in previous studies done by our group.

#### Phenotypic Characterization of Airway Luminal Cells

The functional phenotype of airway luminal T-cells was re-evaluated 2 weeks post-M.tb challenge to understand the immunological basis of the protection induced by MIP. This analysis was undertaken by challenging the mice with M.tb at two time points i.e., 4 weeks (Group 1) and 8 weeks (Group 2) after MIP vaccination. In general, the T-cell response should peak at 2 weeks after infection but M.tb mediated host modulation delays the response by 1 additional week (1). However, in comparison to the only infected “control,” our results demonstrated the presence of significantly higher numbers of CD4^+^ (*p* = 0.032) and CD8^+^ (*p* = 0.021) T_EM_ cells in airway lumen at 2 weeks after M.tb infection in Group 1. The comparative proportion of T_CM_ cells in the lung airway lumen, though overall smaller in number, was observed to be higher in “MIP i.n.” group over that of “MIP s.c.” group (Figure 8D). Similar results were observed when the time interval between immunization and infection was increased to 8 weeks (Group 2), suggestive of persistent T memory response at primary site of M.tb entry following vaccination.

An increasing number of CD69^+^ and CXCR3^+^ T-cells (both CD4^+^ and CD8^+^) were noted in i.n. but not in the s.c. vaccinated group (Figure 8E). It is evident from the above results that immediate Th1 type effector response at pathogen entry site must be playing a role in MIP mediated protection. Lung mRNA expression analysis revealed significant expression of CCL21 post-M.tb infection in MIP i.n. immunized animals as compared to MIP s.c. immunized one (Figure 8F). CCL21 is homeostatic chemokine that assists formation of secondary lymphoid follicles and its expression in M.tb infected lungs has been implicated as a protective biomarker (18).

#### M.tb-Specific Polyfunctional T-Cell Responses

**Figure 9:**
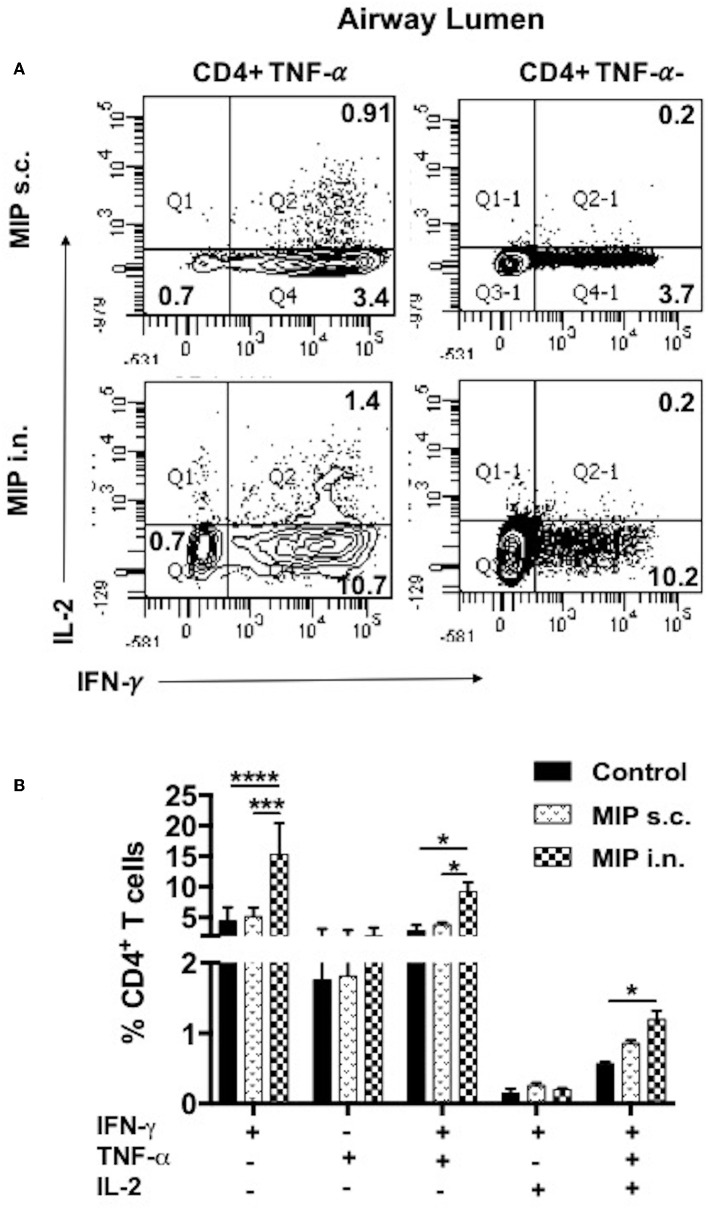
Polyfunctional T-cells after M.tb challenge. T-cells secreting one or more of these cytokines i.e., IFN-γ, TNF-α, and IL-2, were analyzed. Airway luminal cells from all the three groups were isolated 2 weeks post-M.tb challenge (challenged 4 weeks after immunization; Group 1) were re-stimulated with M.tb antigen, stained with antibodies against surface markers and the above cytokines. **(A)** Representative FACS profile polyfunctional CD4^+^ T-cells and **(B)** pooled data of mono- and polyfunctional CD4^+^ T-cells, 2 weeks post M.tb challange. Data is mean ± SEM; analysis was done using two-way ANOVA with Tukey's correction for multiple comparisons. *p ≤ 0.02, ***p ≤ 0.0005, and ****p ≤ 0.0001 (*N* = 2, 10–12 mice/group). *N*, number of experiments.

To examine the status of Ag-specific response post-M.tb challenge, airway luminal cells collected 2 weeks after infection (challenged 4 weeks after vaccination) were stimulated with M.tb whole cell lysate and intracellular staining for cytokines (IFN-γ^+^, TNF-α^+^, and IL-2^+^) was done. The frequency of 3+(IFN-γ^+^, TNF- α^+^, IL-2^+^) and 2+ (IFN-γ^+^, TNF- α^+^) CD4^+^ T-cells were very high in the “MIP i.n.” group as compared to “MIP s.c.” group (Figures 9A,B).

### Intratracheal Transfer of MIP Primed Airway Luminal T-Cells Confers Protection in Naïve Mice

**Figure 10:**
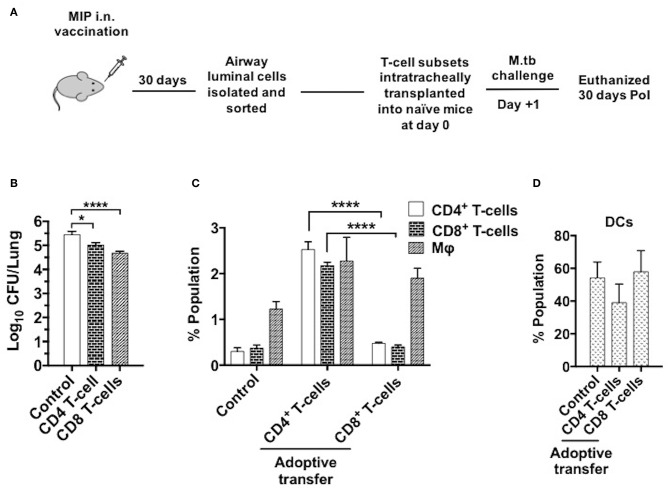
Adoptive transfer of airway-resident T cell populations confers protection against TB. **(A)** T cell subsets were isolated 4–5 weeks following MIP i.n. vaccination and sorted by magnetic-activated cell sorting (MACS). The sorted T cell population purity was assessed as 90–95% (Figure S3). 5 × 10^4^ sorted cells were transferred intratracheally into naive C57Bl/6 mice. One day after the transplant, recipient mice were challenged with M.tb and lung CFU counts were determined 30 days later. **(B)** Bacterial CFU counts in lungs after 30 days. **(C)** Status of T-cells, macrophages and **(D)** DCs in lung airways 30 days post-infection following the intratracheal transfer of sorted CD4^+^ and CD8^+^ T-cells from “MIP i.n.” immunized animals. Data are mean ± SEM and analysis for all the above data was done using one-way ANOVA with Tukey's correction for multiple comparisons. *p ≤ 0.001 and ****p ≤ 0.0001 (*N* = 2, 4 mice/group/experiment). *N*, number of experiments.

To determine the T-cell subpopulation critical for the protection observed following intranasal vaccination, enriched airway luminal CD4^+^/CD8^+^ T-cells (purity upto 90–95%) (Figure S5) were administered into the trachea of naïve mice 1 day prior to M.tb challenge (Figure 10A). Significantly lower bacterial burden was observed in the animals given CD8+ T-cells as compared to saline control, and the reduction observed was ~0.7 log (*p* < 0.0001). While transplantation of CD4^+^ T-cells resulted in a lower bacterial burden of ~0.4 log (*p* = 0.024) mice in lungs. This significant reduction in bacterial load in the group given CD8^+^ T-cells might have been due to the immediate cytolytic activity of CD8^+^ T-cells (Figure 10B). When both CD4^+^ and CD8^+^ subsets (mixed in 1:1 ratio) were transplanted intratracheally, better protection in terms of bacterial burden in lungs was found. A reduction of ~1.0 log in CFU was observed in the group which received a combination of both subsets **(**data not shown). Interestingly, transfer of enriched CD4^+^ T-cells (MIP primed) in naïve mice aided the recruitment of T-cells (CD4^+^ and CD8^+^ subsets; *p* < 0.0001) significantly in lung airways as observed 30 days PoI, in contrast to the transfer of enriched CD8^+^ T-cell (MIP primed). Transfer of both these populations independently led to moderate increase in macrophage percentage (Figure 10C), whereas DCs proportion in airways post-transplantation remained similar after M.tb challenge in vaccinated as well as unvaccinated animals (Figure 10D). Due to low yield of T-cells from airway lumen, sorting various memory subsets and further tracking experiments could not be performed. Though the observations are limited but conclusive. It is evident that MIP primed airway luminal CD4^+^ and CD8^+^ T-cells could adoptively transfer the protection to naïve mice infected with M.tb.

## Discussion

A double blind phase III clinical trial conducted in the 1990s in which MIP was given to the household contacts of leprosy patients (involving about 30,000 people in an area which was endemic for both leprosy and TB) to prevent leprosy. However, the TB incidence was also found to be reduced significantly as compared with that of the placebo group after 13 years in retrospective analysis (19).

This encouraging observation led to further investigation of MIP as a potential vaccine candidate against TB. Protection studies led by our group demonstrated MIP, as whole cell vaccine, to be more efficacious than BCG vaccine in animal models of TB (20). It conferred better protection when administered by nasal route, in both live and killed forms (5). The enhanced protective efficacy of MIP can be attributed to its unique immunogenic properties. Route of vaccination matching the route of infection is stated to be more logical for direct mounting of a memory response in target organ (21). However, our knowledge of detailed mechanisms of protective immune response elicited in different lung compartments i.e., airway lumen and lung interstitium following intranasal MIP vaccination still needs further investigation. The present study suggested that effective lung airway luminal immune response induced by MIP i.n. vaccination could be a key player in improved protection observed previously (5, 6).

In general, the human immune system is capable of controlling M.tb infections. Immune activation and cellular trafficking between the lungs and lymph nodes post-infection result in accumulation of activated T-cells and leukocytes in lung tissue, and confines M.tb infected macrophages in the granuloma (22). However, M.tb has adopted several mechanisms to initially delay this process of cell migration/positioning so as to multiply in number. The restriction of M.tb growth at a very early stage of infection thus necessitates prior presence of antigen-specific immune cells at the entry site (alveolar mucosa), that can swiftly respond to future infection(s) (21).

In the current study it was noted that MIP immunization via i.n. route (but not by s.c. route) skewed the immune cell equilibrium of alveolar lumen toward T-cells (both CD4^+^ and CD8^+^ subsets) and a similar trend was observed at 2 weeks post-M.tb challenge also. Cellular homeostasis of alveolar luminal compartment was maintained in MIP vaccinated groups because the increase in T-cell percentage was followed by a moderate decrease in the number of APCs (macrophages, DCs and NK-cells). However, after infection, macrophage population in the lungs increased in the “MIP i.n.” group, compared with “MIP s.c.” group. This increase could have been due to the prior presence of memory T-cells which upon stimulation facilitated the recruitment of macrophages to the site (lung airways) following M.tb exposure. Stating the relative significance of CD4^+^ and CD8^+^ subsets in M.tb infection is difficult because the vaccine-mediated protective potential of CD4^+^ T-cells is counted on its ability to activate APCs and CD8^+^T-cells for its direct killing mechanism (23).

The current study presents detailed analysis of various T-cell subsets, as a mere increase in the number of T-cells does not justify protective immunity. CD4^+^ and CD8^+^ T-cells in intranasal MIP vaccinated mice (both before and after M.tb challenge) can be further divided into subsets depending on the surface expression of CD44 and CD62L. T_EM_ cells (both CD4^+^ and CD8^+^) with CD44^+^ CD62L^−^ phenotype were very high in number in the airway lumen compartment following nasal MIP vaccination as compared to s.c. immunization. These are subset of memory T-cells generated in recently infected peripheral tissue (lung in this case); they live longer than T_Eff_ cells, but shorter-lived than T_CM_ cells. T_EM_ lacks lymph node homing marker thus persists and imparts immunity at peripheral sites while T_CM_ cells tend to home to secondary lymphoid organs and are supposed to be present in smaller numbers at peripheral sites. In this study, the proportion of T_CMs_ was far smaller than T_EMs_ but, interestingly, the airway lumen of “MIP i.n.” group harbored comparatively a higher number of T_CM_ cells (CD44^+^ CD62L^+^) as compared to the “MIP s.c.” group, which suggested the presence of antigen-specific long term memory in this compartment as well. In the lung interstitium compartment also at later time point after immunization (8 week), comparatively higher T_CM_ and T_EM_ populations were found in “MIP i.n.” than in the ‘MIP s.c.” group. The observation also highlighted the basic fact that s.c. route is leading to suboptimal immune response in lungs. Interestingly, T_CM_ cells in the spleen of “MIP i.n.” and “MIP s.c.” groups were found to be similar (more than in the “unimmunized control”), providing evidence of the advantage of intranasal vaccination in mounting local and systemic response thus, claiming an improved protection over s.c. route (24). During subsequent exposure to M.tb, these T_EM_ and T_CM_ cells would undergo a rapid multiplication resulting in a more robust recall response than a primary response. In an adoptive transfer study in non-human primate model, clones of CD8^+^ T_CM_ cells provided long-term *in-vivo* protection (25). In another immunotherapeutic study, CD4^+^ memory T-cells were shown to support the development of CD8^+^T-cell memory function, thereby highlighting the importance of the presence of both these subsets in lungs for protection against M.tb (26).

A very important observation from previous studies with MIP is that it is cleared off from the host body in 4–6 weeks. This resolution of infection and inflammation might be the reason behind the generation of a “true” memory response, observed as the persistent T_CM_ population in airway lumen. Failure of the BCG vaccine to achieve sufficient T_CM_ response might be due to the prolonged antigen presence, which could be partially responsible for its failure to protect against pulmonary TB in adults (27–29). Our post-M.tb challenge study demonstrated that these memory cells generated strong and immediate effector responses in the “MIP i.n.” group, which could be responsible for the enhanced protection observed in previous studies.

A potent T_CM_ response post-MIP vaccination also corresponded with a strong T_EM_ response post-M.tb challenge. Migration of these cells to the mucosal site requires chemokine receptor-ligand axis. CXCR3 expression is considered critical for recruitment of T-cells to site of infection (30). Expression of CXCR3 by a significant proportion of T-cells in airway lumen suggested cellular migration via CXCR3-CXCL11 axis following MIP i.n. vaccination. Reportedly, Th1 committed effector T-cell subsets with up-regulated expression of CXCR3 differentiate to effector and central memory cells and has been implicated in control of M.tb (8, 9, 31). In systemically immunized hosts, these cells remains in the lung vasculature, failing to populate lung mucosa because local lung inflammation is required to trigger expression of chemo-attractants to facilitate site specific cellular migration (9). A minimal proportion of T-cells expressing chemokine receptors CXCR4, CXCR5 and CCR7 were observed in the lung airway lumen, till 8 weeks of follow-up. CXCR4 and CXCR5 are preferentially expressed by Th2 cells, helping in development and positioning of B-cells (32). Reports have suggested role of CXCR4 in supporting M.tb survival inside the granuloma. CXCR4 is a known pro-angiogenesis factor helping vascularization of granuloma thus facilitating supply of oxygen and nutrients deep inside it (33). Our findings from chemokine receptor-ligand profiling before infection thus confirms induction of Th1 but no sign of Th2 response in mice.

In “post-M.tb challenge” studies, increased expression of chemokine ligands CCL21 in lungs was observed in “MIP i.n.” vaccinated group. CCL21 is a homeostatic chemokine ligand implicated in TB protection due to its assistive role in tertiary lymphoid follicles formation in lungs which helps in proper granuloma organization and accumulation of antigen-specific IFN-γ producing T-cells at the site of infection. M.tb infection is known to perturb expression of this ligand by lung endothelial cells, which in turn hampers development of an organized lymphoid follicles in lungs, thus successfully supporting its proliferation (18).

MIP intranasal vaccination results in significant recruitment of T-cells in airway lumen and also in lung interstitium to some extent. *Ex-situ* stimulation of cells isolated from both compartments of lung with M.tb whole cell lysate provided evidence of the presence of a higher number of proliferative cells in the “MIP i.n.” group. The results also suggested one of the major drawbacks of MIP s.c. vaccination which, although capable of eliciting antigen-specific T-cells that populate lung via the pulmonary vasculature, fails to induce their homing into airway lumen. T-cells residing in lung vascular bed following s.c. vaccination depends on M.tb-loaded APCs to migrate to them for activation and proliferation, thus delaying the effector response (27).

Memory T-cells induced by MIP were found to be functionally heterogeneous. The enhanced protection proffered by nasal MIP administration could be attributed to the memory cells present at the primary site of infection, which produce Th1- and Th17- type cytokine responses as soon as they encounter M.tb. This was evident from the present findings; MIP induced cells in pulmonary and systemic compartments secreted IFN-γ, IL-2, IL-17, IL-12, and TNF-α on *ex-vivo* stimulation with M.tb antigens. Th1 and Th17 lineages have been suggested to be crucial mediators of protective response in TB (10); Th1 cytokines are essential in controlling the growth of M.tb. IFN-γ secreting T-cells activate M.tb infected macrophages and DCs (34). IL-12 induces transcription factors signal transducer and activator of transcription-4 (STAT4) and T-bet, which are known to be important for IFN-γ production. Another important cytokine, TNF-α, is a key player in the maintenance of granuloma and is also believed to synergize with IFN-γ to mediate M.tb clearance (35, 36). The IL-17 producing T cells observed in the airway lumen could be contributing to protection by recruitment of neutrophils at the site of infection during acute phase. Vaccine induced Th17 cells are reportedly involved in memory response against future M.tb infections and thus are considered indispensable for protection against TB (10). A panel of cytokines secreted by MIP induced memory T cells could be responsible for orchestrating effector response against M.tb challenge.

Furthermore, the quality of T-cells on per cell basis was studied for their ability to simultaneously secrete more than one cytokine i.e., IFN-γ, TNF-α, and IL-2. Mice vaccinated with MIP by intranasal route had higher frequency of M.tb specific polyfunctional T-cells (predominantly CD4^+^ subset) in airway mucosa of lung, compared with “MIP s.c.” group. Further analysis of polyfunctional T-cell responses in different setups– “MIP vaccinated,” “MIP vaccinated + M.tb challenged,” and “only M.tb challenged” animals led to the conclusion that airway lumen of “MIP i.n.” vaccinated animals before infection had a significantly high proportion of 2^+^ (IFN-γ^+^ IL-2^+^) CD4^+^ T-cells which represent memory cells, but these were not observed post-M.tb challenge. This might be due to the conversion of Ag-specific memory cells to effector population which starts secreting TNF-α on antigen encounter. Reports suggest the functional superiority of multifunctional T cells over their single cytokine producing counterparts (15, 16). In tuberculosis, the role of multifunctional CD4^+^ T cells has been well-elaborated, but the role of their CD8^+^ counterparts is less clear (37).

Evaluation of the protective potential of lung airway luminal Th1 cells was done by adoptive transfer of T-cell subsets isolated from this compartment of “MIP i.n.” group into naïve mice followed by M.tb challenge. Transfer of enriched CD8^+^ T-cells notably led to a higher reduction in M.tb burden in the lungs as compared to CD4^+^ T-cells which suggest that MIP primed CD8^+^ T-cells were efficient in direct killing of pathogen and the infected host cells resulting in comparatively lower M.tb load. The immune cell analysis, on the other hand, revealed significantly increased proportions of both T-cell subsets in animals receiving CD4^+^ but not CD8^+^ T-cells (at that time point); but it was interesting to note that both the subsets could recruit macrophages at the site. This phenomenon is strongly supported by the fact that CD8^+^ memory cells, together with CD4^+^ memory T-cells, secrete IFN-γ and TNF-α which activate macrophages and help in their recruitment. Also, due to cellular lysis induced by CD8^+^ T-cells, an inflammatory environment is created that in turn recruits more macrophages to the vicinity. Here CD4^+^ T-cells lag behind in immediate bacterial load reduction due to the absence of direct killing mechanisms but are nevertheless indispensable for long term protective immunity (38, 39).

## Conclusions

Conclusively, our results identify correlates of protective response induced by MIP, against TB, in mice model. Specifically, the current data suggests that MIP given by nasal route more effectively induce homing of antigen-specific Th1 cells in the lung airways which s.c. route of immunization fails in.

## Data Availability Statement

All datasets generated for this study are included in the manuscript/Supplementary Files.

## Ethics Statement

This study was carried out in accordance with the principles of the Basel Declaration and recommendations of Institutional Animal Ethics Committee of National Institute of Immunology. The protocol was approved by the above committee under serial number IAEC#434/17.

## Author Contributions

AG and SB contributed to study design, data analysis, and data interpretation. AG, MS, and AN have performed the experiments. AG, SB, BS, and LP have contributed to manuscript preparation. AG and SB have read and approved the final manuscript.

### Conflict of Interest

The authors declare that the research was conducted in the absence of any commercial or financial relationships that could be construed as a potential conflict of interest.

## Supplementary Material

Figure S1 Percentage of total leucocytes in pulmonary and systemic compartments at 1 week and 4 weeks post-immunization were accessed. Mean percentage leucocytes in **(A)** airway lumen, **(B)** lung interstitium, and **(C)** spleen of immunized and unimmunized mice. Data are mean ± SEM; analysis was done using two-way ANOVA with Tukey's correction for multiple comparisons (*N* = 3, 8–10 mice/group/experiment). *N*, number of experiments.

Figure S2 Transcription factor Ki67 was measured at mRNA level by RT-PCR of lungs before and after MIP vaccination. Data is mean ± SEM of 6 mice from two independent set of experiments; analysis was done using one-way ANOVA with Tukey's correction for multiple comparisons. ^*^*p* ≤ 0.05.

Figure S3 Memory T-cell subpopulations in spleen after vaccination. Effector (CD44+ CD62L–) and central (CD44+ CD62L+) memory T-cells 4 and 8 weeks post-MIP vaccination in spleen. Data are mean ± SEM; analysis was done using two-way ANOVA with Tukey's correction for multiple comparisons. ^*^*p* ≤ 0.05 (*N* = 3, 8–10 mice/group/experiment). *N*, number of experiments.

Figure S4 Mycobacterial antigen-specific memory recall response in splenocytes following incubation with M.tb whole cell lysate. The culture supernatants were collected 24 and 48 h after treatment. Proinflammatory cytokines-IL-2, IL-17, IFN-γ, IL-12p40, TNF-α, and anti-inflammatory cytokine IL-10 were analyzed by ELISA. Data are mean ± SEM; analysis was done using one-way ANOVA with Tukey's correction for multiple comparisons. ^*^*p* ≤ 0.05 (*N* = 3, 8–10 mice/group / experiment). *N*, number of experiments.

Figure S5 Purity assessment of T-cell subsets after MACS sorting. Percentage purity of MACS sorted airway luminal CD4^+^ and CD8^+^ T-cells was evaluated by FACS analysis on the basis of their respective surface markers. Ninety to ninety-five percent pure cells population were used for intratracheal transplantation.

Figure S6 Representative FACS profile of airway luminal T-cells expressing chemokine receptors CXCR3, CXCR4, CXCR5, and activation marker CD69 for both **(A)** CD4^+^ and **(B)** CD8^+^ subsets, 8 weeks after MIP vaccination. CD4+ and CD8+ T-cells were gated and drilled down separately for all these markers.

## Abbreviations

Ag: antigen
i.n.: intranasal
MIP: *Mycobacterium indicus pranii*
M.tb: *Mycobacterium tuberculosis*
TB: tuberculosis
T_CM_: central memory T-cells
T_EM_: effector memory T-cells
PoI: post-infection
s.c.: subcutaneous.
