# Supplementary figures and images for: Mycobacterium indicus pranii Induced Memory T-Cells in Lung Airways Are Sentinels for Improved Protection Against M.tb Infection
Source: Front Immunol. 2019 Oct 18;10:2359. doi: 10.3389/fimmu.2019.02359 (PMC6813244; doi:10.3389/fimmu.2019.02359)

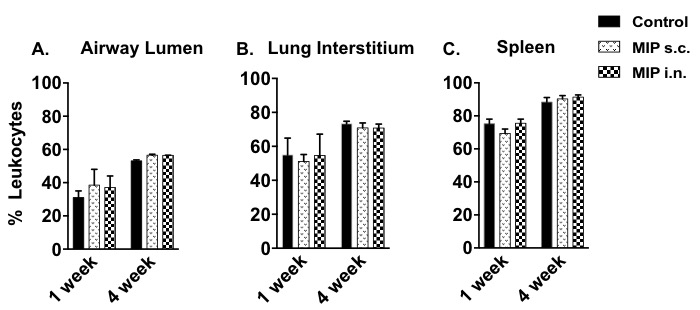

Supplement: Figure S1 — Percentage of total leucocytes in pulmonary and systemic compartments at 1 week and 4 weeks post-immunization were accessed. Mean percentage leucocytes in (A) airway lumen, (B) lung interstitium, and (C) spleen of immunized and unimmunized mice. Data are mean ± SEM; analysis was done using two-way ANOVA with Tukey's correction for multiple comparisons (N = 3, 8–10 mice/group/experiment). N, number of experiments. [file Image_1.JPEG]

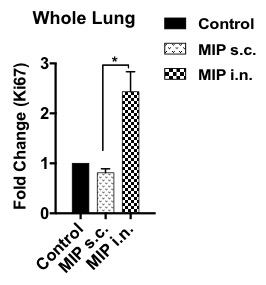

Supplement: Figure S2 — Transcription factor Ki67 was measured at mRNA level by RT-PCR of lungs before and after MIP vaccination. Data is mean ± SEM of 6 mice from two independent set of experiments; analysis was done using one-way ANOVA with Tukey's correction for multiple comparisons. *p ≤ 0.05. [file Image_2.JPEG]

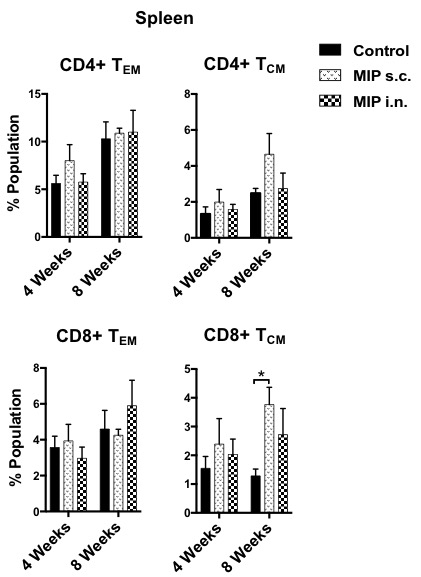

Supplement: Figure S3 — Memory T-cell subpopulations in spleen after vaccination. Effector (CD44+ CD62L–) and central (CD44+ CD62L+) memory T-cells 4 and 8 weeks post-MIP vaccination in spleen. Data are mean ± SEM; analysis was done using two-way ANOVA with Tukey's correction for multiple comparisons. *p ≤ 0.05 (N = 3, 8–10 mice/group/experiment). N, number of experiments. [file Image_3.JPEG]

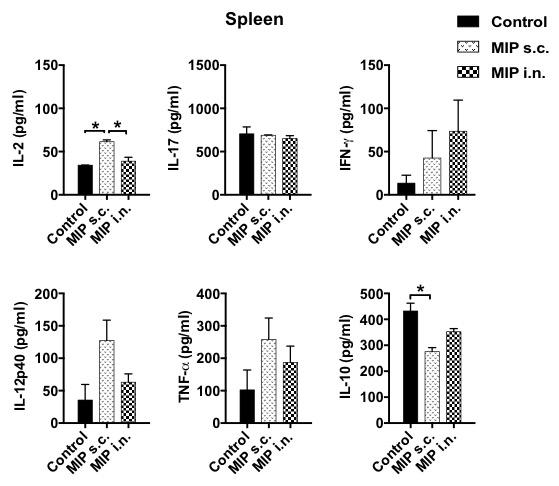

Supplement: Figure S4 — Mycobacterial antigen-specific memory recall response in splenocytes following incubation with M.tb whole cell lysate. The culture supernatants were collected 24 and 48 h after treatment. Proinflammatory cytokines-IL-2, IL-17, IFN-γ, IL-12p40, TNF-α, and anti-inflammatory cytokine IL-10 were analyzed by ELISA. Data are mean ± SEM; analysis was done using one-way ANOVA with Tukey's correction for multiple comparisons. *p ≤ 0.05 (N = 3, 8–10 mice/group / experiment). N, number of experiments. [file Image_4.jpg]

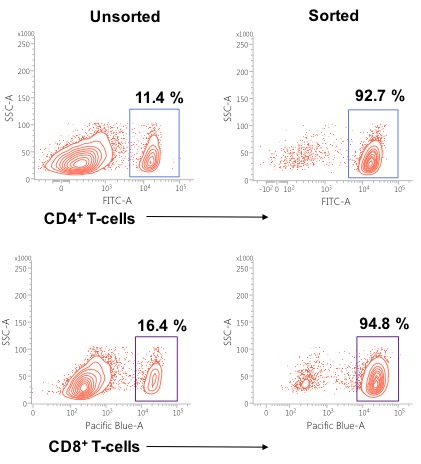

Supplement: Figure S5 — Purity assessment of T-cell subsets after MACS sorting. Percentage purity of MACS sorted airway luminal CD4+ and CD8+ T-cells was evaluated by FACS analysis on the basis of their respective surface markers. Ninety to ninety-five percent pure cells population were used for intratracheal transplantation. [file Image_5.JPEG]

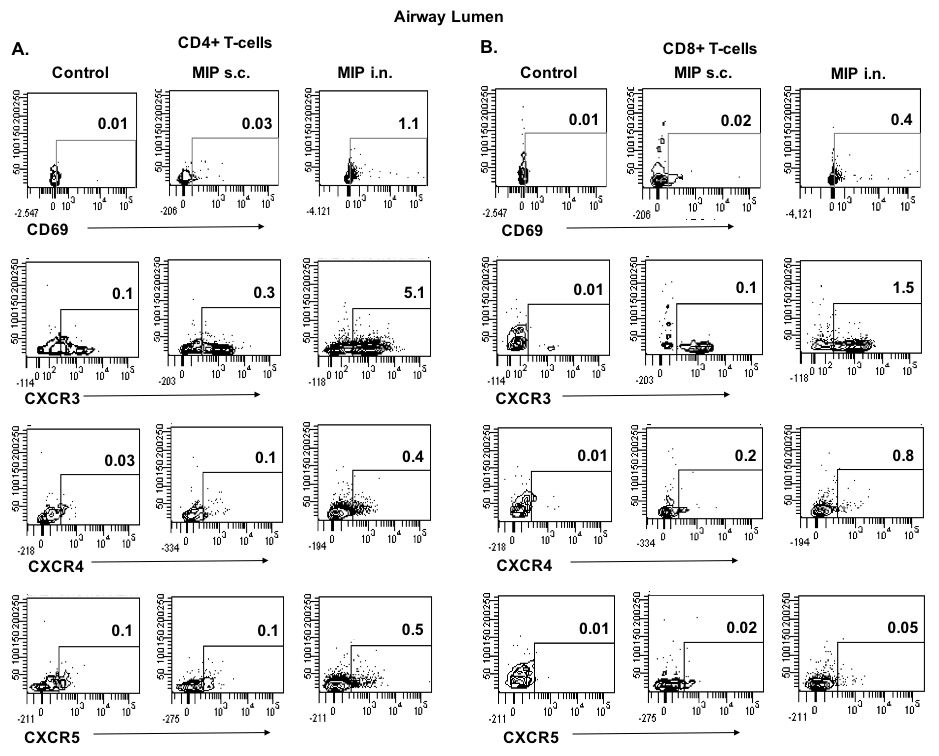

Supplement: Figure S6 — Representative FACS profile of airway luminal T-cells expressing chemokine receptors CXCR3, CXCR4, CXCR5, and activation marker CD69 for both (A) CD4+ and (B) CD8+ subsets, 8 weeks after MIP vaccination. CD4+ and CD8+ T-cells were gated and drilled down separately for all these markers. [file Image_6.JPEG]
